# New nursing roles for the integrated management of complex chronic and palliative care patients in the region of Valencia/Nuevos perfiles enfermería para el manejo integral de pacientes crónicos complejos y paliativos en la Comunidad Valenciana

**Published:** 2012-05-29

**Authors:** Juan Gallud, Pepa Soler, Dolores Cuevas

**Affiliations:** General Management Office for the Healthcare Planning and Provision, Valencia Health Agency, Valencia Health Ministry, Valencia, Spain; General Management Office for the Healthcare Planning and Provision, Valencia Health Agency, Valencia Health Ministry, Valencia, Spain; General Management Office for the Healthcare Planning and Provision, Valencia Health Agency, Valencia Health Ministry, Valencia, Spain

**Keywords:** nurses, case management, continuity of care, integrated care, home care services, hospital at home, enfermera, gestión de casos, continuidad atención integrada, atención domiciliaria, hospital a domicilio

## Introduction

The autonomous region of Valencia has a population of 5.1 million divided into 24 health districts and the governing body of the Valencia Health Service is the Valencia health agency (AVS*, Agència Valenciana de Salut*). In the framework of the plan for improving home care in the region of Valencia [1] a study was carried out between 2005 and 2006, across three of these health districts. The study assessed the quality of home care provided by the two largest agents (by volume of activity): the primary care teams (PCTs—a total of 241, at present) through a home care programme (HCP) and the hospital-at-home (HaH) units, well developed in the AVS region (with a total of 24, at present). Able to attend 1428 patients per day, these HaH units supported 35,802 discharges in 2010. The main objective of the study was to establish a basis for designing improvement interventions in the sphere of home care. Notably, the results demonstrated a fragmentation and lack of continuity of care between HaH units and the HCP for palliative care patients and those with advanced chronic diseases, a very complex group of patients associated with a high use of resources that may jeopardise the sustainability of the system.

**Figure 1.**
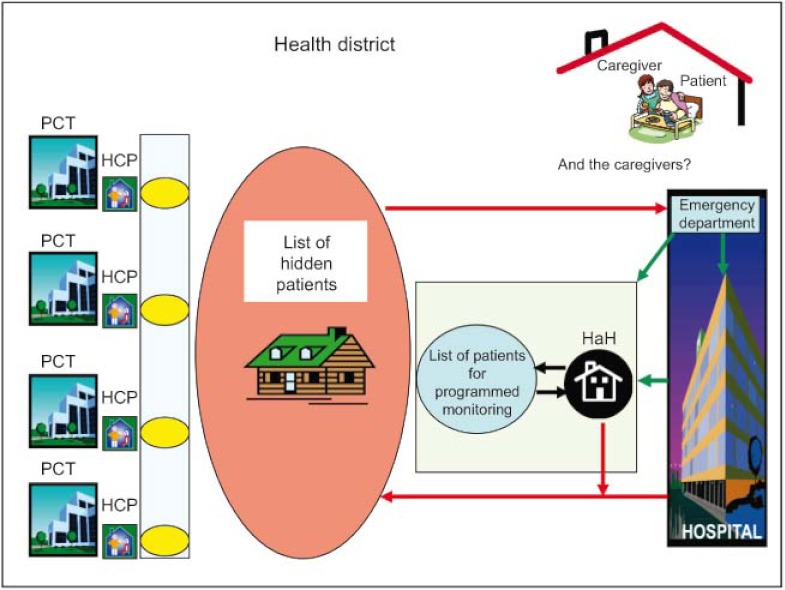
Assessment of the home care programme (HCP) and hospital at home (HaH) units in 2005–6: fragmentation and lack of continuity of care in the field of home care. PCT: Primary Care Team. HCP: Home care programme run by PCTs. HaH: Hospital at Home Unit.

On discharge from the HaH units, the low level of development of the HCP meant that there were difficulties in monitoring patients at home. Given this, the response of the HaH units to the most complex patients was to create a “list of patients for programmed monitoring” that they handled alone, independently of the PCTs. All other patients on discharge from an HaH unit or hospital joined a “list of hidden patients” since, as the PCTs were not aware of their need for home care, they were not included in the HCP and their care was not under any type of monitoring or control. Moreover, caregivers were not considered eligible for support by health professionals (Figure 1).

## Description of the project

Given the situation described, the search for solutions was focused on nursing staff, key professionals in the field of home care, and on improvements in information and communication technologies (ICTs) and the use thereof. Following the recommendations of a group of experts, and on the basis of other pilots carried out in Spain [2–4] and other countries [5–12], two new nursing roles were introduced, namely:

- Nurse community case managers (NCCMs).
- Hospital liaison nurses (HLNs).

### Target population

The target population of the NCCMs and the HLNs are complex cases, most of which are eligible for home care, that is, individuals at the apex of the Kaiser Permanente pyramid [8]. The term case was used to refer to a group formed by a patient, caregiver and their environment. We included patients with advanced chronic conditions in a broad sense: organic and mental illness, those being treated to cure and those at the end stage of diseases, and both adult and paediatric populations. The level of complexity of each case is determined by the combination of the complexity of the clinical and community management.

### Mission and functions

Their mission is to guarantee integrated and continuous care for complex cases. Their core functions include: active searching, case management, linkage between care sites and support to caregivers. The nurses with these two roles work together and have similar functions but in different spheres: the NCCMs work in the community, while the HLNs work in a hospital environment (conventional hospital or HaH).

### ICTs

The nurses were provided with all suitable ICTs. In particular, the electronic medical records were used as a work tool and for communication between NCCMs and HLNs. Further, we developed a computer program that identifies potential complex patients from hospital databases.

In March 2007, we started a pilot in two health districts (Castellón and Alicante) with the involvement of 3 HaH units, 10 PCTs, 3.5 HLNs and 9 NCCMs. The catchment population of the NCCMs in the two districts is 210,689 (39% of the total population) though not all the PCTs had NCCMs. The data presented here were collected over a period of four years, until March 2011. However, these nurses only became involved in the palliative care pathways in 2009 and, accordingly, the data concerning this group of patients covers only a 2-year period (2009 and 2010).

## Results

The key results are shown in Table 1. In terms of outcomes, the introduction of the HLNs and NCCMs has contributed to:

- Significantly increasing the identification of cases by active searching, using both the usual and the new electronic method, and ensuring that the most complex cases are monitored (2052).
- Promoting home care by PCTs, reaching an average coverage of 58% from 15% at baseline, and reducing the number of “hidden patients” and number of patients on the list for “programmed monitoring”.
- Improving continuity of information by use of shared electronic medical records.
- Improving continuity of care, thanks to the large number of bidirectional links between PCTs and HaH units instigated by these nurse.
- Improving healthcare integration: the close interaction achieved by these links has reinforced the supportive role of HaH units to PCTs and the provision of shared care, increasing the ability to solve problems at home and replacing potential admissions to hospital by transfers of care to HaH units, with the associated benefits for patients and caregivers, as well as in terms of the efficiency of the system.
- Promoting new services: telephone health services and group activities.
- Achieving rates of home death of 67%, with the inclusion of palliative care patients on their list of patients.
- Significantly reducing the use of emergency services (–77%) and hospital admissions (–70%) of patients under their care over a year compared to the year prior to their assessment, the figures being –78% and –64%, respectively, for palliative care patients.
- Turning caregivers into new “users” of the system.

## Conclusions

The introduction of two new nursing roles, NCCMs and HLNs, to the home care setting, whose main agents in the AVS are PCTs and HaH units, and the enhanced use of ICTs have contributed to improving care for complex chronic and palliative care patients. In particular, they have proven to be essential roles for identifying such patients and achieving integration and continuity of care.

By the end of 2011, the deployment of the AVS model will be extended to 8 districts, with the involvement of 8 HaH units, 26 PCTs, 9 HLNs and 25 NCCMs, reaching a catchment population of 520,000 (10% of the autonomous region of Valencia).

## Conference abstract Spanish

## Introducción

La Comunidad Valenciana (CV) tiene 5,1 millones de habitantes y está dividida territorialmente en 24 departamentos de salud. La Agencia Valenciana de Salud (AVS) es el organismo central de gobierno del servicio de salud. En el marco del Plan para la Mejora de la Atención Domiciliaria de la CV [1] se realizó, entre 2005 y 2006, un estudio en 3 departamentos para evaluar la atención domiciliaria prestada por los dos agentes con más volumen de actividad: los Equipos de Atención Primaria (EAP, 241 actualmente) a través del programa de atención domiciliaria (PAD) y las Unidades de Hospital a Domicilio (UHD), una prestación muy desarrollada en la AVS con 24 UHD, capacidad para atender a 1.428 pacientes al día y 35.802 altas en 2010. El objetivo principal era establecer las bases para intervenciones de mejora en el ámbito de la atención domiciliaria.

Los resultados ilustraron la fragmentación y la falta de continuidad de la atención entre UHD y PAD para los pacientes crónicos avanzados y paliativos, un grupo de pacientes de alta complejidad y de gran consumo de recursos que puede amenazar la sostenibilidad del sistema.

Al alta de las UHD, el escaso desarrollo del PAD generaba dificultades de seguimiento domiciliario. Ante tal situación, la respuesta de las UHD para los pacientes más complejos fue crear una “bolsa de seguimiento programado” totalmente a su cargo y al margen de los EAP.

El resto de enfermos al alta de la UHD o del Hospital engrosaba una “bolsa de pacientes ocultos” ya que, al ser desconocida su condición domiciliaria por los EAP, no eran incluidos en el PAD y su atención quedaba fuera de cualquier tipo de programación y control.

Los cuidadores no eran considerados objetivo de apoyo por los profesionales sanitarios (Figura 1).

## Descripción de la experiencia

Ante la situación descrita, la búsqueda de soluciones se orientó hacia el colectivo de enfermería, profesionales muy relevantes en la atención domiciliaria, y hacia la mejora de las tecnologías de la información y comunicación (TIC). Siguiendo las recomendaciones de un grupo de expertos, y en base a experiencias tanto nacionales [2–4] como internacionales [5–12], se diseñaron dos nuevos perfiles de enfermería adaptados a nuestra realidad:

- Enfermeras de Gestión Comunitarias (EGC).
- Enfermeras de Enlace Hospitalarias (EEH).

### Población diana

La población diana de las EGC-EEH son los casos complejos, la mayoría susceptibles de atención domiciliaria y que, conceptualmente, se encontrarían en el vértice de la pirámide de Kaiser [8]. Se definió como “caso” al conjunto formado por paciente, cuidador y entorno de vida. Como pacientes, se incluyen enfermos crónicos avanzados en sentido amplio, abarcando enfermedades tanto orgánicas como mentales, en fase curativa o de final de vida y tanto población adulta como pediátrica. El nivel de complejidad de cada caso viene determinado por la combinación de la complejidad de manejo clínico y comunitario.

### Misión y funciones

Su misión es garantizar una atención integrada y continua de los casos complejos.

Sus funciones nucleares son: búsqueda activa; gestión de casos; enlace entre ubicaciones asistenciales; y apoyo a cuidadores. Ambos perfiles profesionales trabajan conjuntamente y tienen funciones similares pero desarrolladas en ámbitos distintos: las EGC en el comunitario y las EEH en el hospitalario (hospital convencional y hospital a domicilio).

### TIC

Se ha dotado a las enfermeras con todas las TIC disponibles.

Se ha utilizado la historia clínica electrónica como instrumento de trabajo y de comunicación entre EEH y EGC.

Se ha desarrollado una aplicación informática que, explorando bases de datos hospitalarias, permite identificar pacientes potencialmente complejos.

En marzo de 2007 se inició un pilotaje en dos departamentos de salud (Castellón y Alicante). Participaron 3 UHD, 10 EAP, 3,5 EEH y 9 EGC. La cobertura poblacional de las EGC en ambos departamentos fue de 210.689 habitantes (39% de su población). Había EAP con y sin EGC. Los datos presentados abarcan 4 años hasta marzo 2011. En 2009, estas enfermeras se incorporaron a los circuitos de atención paliativa, los datos para este colectivo incluyen 2 años, 2009 y 2010.

## Resultados

Los resultados más relevantes se presentan en la tabla 1. En cuanto a los resultados, la implantación de EEH-EGC ha contribuido a:

- Aumentar significativamente la captación de casos mediante la búsqueda activa por medios habituales y/o electrónicos, y el poner bajo control a los casos más complejos (2.052).
- Dinamizar la atención domiciliaria de los EAP, alcanzando una cobertura media del 58% desde un 15% inicial, reduciendo las bolsas de pacientes ocultos y de seguimiento programado.
- Mejorar la continuidad informativa mediante la utilización conjunta de la historia clínica electrónica.
- Mejorar la continuidad asistencial gracias al gran volumen de enlaces bidireccionales entre EAP y UHD gestionados por estas enfermeras.
- Mejorar la integración asistencial: la fuerte interacción conseguida por los enlaces ha reforzado la función de soporte de las UHD hacia los EAP y la atención compartida, aumentando la capacidad de resolución de problemas en domicilio y sustituyendo probables ingresos hospitalarios por ingresos en UHD, con las consiguientes ventajas tanto para pacientes y cuidadores como para la eficiencia del sistema.
- Impulsar nuevas prestaciones: la atención telefónica y grupal.
- Incorporar a los enfermos paliativos en su cartera de pacientes y lograr una mortalidad en domicilio del 67%.
- Reducir sustancialmente el consumo anual de urgencias (–77%) e ingresos hospitalarios (–70%) de los pacientes bajo su control en relación a los 12 meses previos a su valoración. En paliativos, –78% y –64% respectivamente.
- Convertir a los cuidadores en nuevos “clientes” del sistema.

## Conclusiones

La incorporación de dos nuevos perfiles enfermería (EGC y EEH) al circuito de atención domiciliaria, cuyos principales agentes en la AVS son los EAP y las UHD, y el desarrollo de las TIC, ha contribuido a mejorar la asistencia a pacientes crónicos complejos y paliativos, convirtiéndose en elementos fundamentales para su captación y para la integración y la continuidad de su atención.

Al final de 2011 el despliegue del modelo en la AVS alcanzará a 8 departamentos, con 8 UHD, 26 EAP, 9 EEH y 25 EGC y cubrirá a una población de 520.000 habitantes (10% de la CV).

## Figures and Tables

**Table 1.**
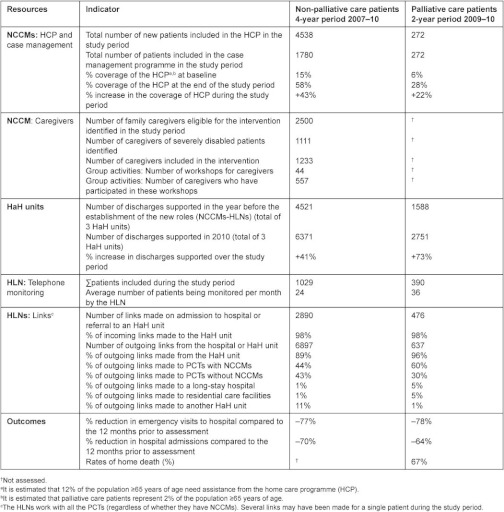
Establishment of 9 nurse community case managers (NCCMs) and 3.5 hospital liaison nurses (HLNsdata on their activity and outcomes for non-palliative care and palliative care patients
